# Comparisons of resting-state brain activity between insomnia and schizophrenia: a coordinate-based meta-analysis

**DOI:** 10.1038/s41537-022-00291-3

**Published:** 2022-10-07

**Authors:** Ziyang Gao, Yuan Xiao, Ye Zhang, Fei Zhu, Bo Tao, Xiangdong Tang, Su Lui

**Affiliations:** 1 Huaxi MR Research Center (HMRRC), Department of Radiology, West China Hospital of Sichuan University, Chengdu, China; 2 Sleep Medicine Center, Department of Respiratory and Critical Care Medicine, Mental Health Center, Translational Neuroscience Center, and State Key Laboratory of Biotherapy, West China Hospital of Sichuan University, Chengdu, China

**Keywords:** Schizophrenia, Schizophrenia

## Abstract

Growing evidence shows that insomnia is closely associated with schizophrenia (SCZ), but the neural mechanism under the association remains unclear. A direct comparison of the patterns of resting-state brain activities would help understand the above question. Using meta-analytic approach, 11 studies of insomnia vs. healthy controls (HC) and 39 studies of SCZ vs. HC were included to illuminate the common and distinct patterns between insomnia and SCZ. Results showed that SCZ and insomnia shared increased resting-state brain activities in frontolimbic structures including the right medial prefrontal gyrus (mPFC) and left parahippocampal gyrus. SCZ additionally revealed greater increased activities in subcortical areas including bilateral putamen, caudate and right insula and greater decreased activities in precentral gyrus and orbitofrontal gyrus. Our study reveals both shared and distinct activation patterns in SCZ and insomnia, which may provide novel insights for understanding the neural basis of the two disorders and enlighten the possibility of the development of treatment strategies for insomnia in SCZ in the future.

## Introduction

Schizophrenia(SCZ) is a chronic and severe mental disorder, manifested as positive symptoms such as hallucinations and delusions, negative symptoms like social withdrawal and apathy, and cognitive symptoms including deficits in working memory and executive function^1^. Although not included in the diagnostic criteria of schizophrenia, accumulating evidence has revealed that insomnia, one of the most prevalent health complaints worldwide^2^, is closely associated with schizophrenia^3–6^. Insomnia can be a predictive factor to estimate the probability that people at high risk develop into psychosis^7^. Moreover, insomnia plays an important role in exacerbating the core symptoms of schizophrenia, such as cognitive impairments^8,9^, auditory hallucinations and delusions^10,11^, social withdrawal and depression^12^. Clinical trials show an exciting prospect that treatment of insomnia may minimize the impact of psychotic symptoms and improve quality of life^13,14^. However, the neurobiological underpinnings for the pathogenesis of schizophrenia and insomnia are yet to be illuminated.

Several neurochemical studies^5,15^ have sought to elucidate the mechanism behind schizophrenia comorbid insomnia from different perspectives. For example, the hyperactivity of D2 receptors in the striatum^16^, reduction of GABA activity^16^, and lower levels of glutamate^17^ can disrupt circadian rhythm and increase wakefulness, ultimately leading to insomnia. Some researchers found that the deficits of sleep spindle activity, which is a representative thalamocortical oscillation, were observed in insomnia patients and also relate to the cognitive impairments in patients with schizophrenia^18–20^. However, it remains unclear whether insomnia and schizophrenia share similar deficits in neural networks.

Over the past decades, resting-state functional MRI (rs-fMRI), a neuroimaging technique that has provided a noninvasive and task-free method to explore intrinsic brain activity and functional connectivity in brain networks^21^, has been applied widely in both insomnia and schizophrenia to explore the neuropathological abnormalities behind^22–24^. The regional spontaneous brain activity can be commonly measured by three derived indices of rs-fMRI: the amplitude of low-frequency fluctuations (ALFF), fractional ALFF (fALFF), and regional homogeneity (ReHo) of the blood-oxygen-level-dependent (BOLD) signals^25,26^. ALFF and fALFF algorithms can measure the total power of the low-frequency BOLD signal, which is relevant to regional neural activity. Meanwhile, ReHo analysis reflects the synchrony of regional brain activity^26^. Although the principles and algorithms among these indices are different, all of them could reflect regional spontaneous brain activities in essence and their combination offers a more comprehensive assessment of brain dysfunction^27,28^. The decreased intrinsic brain activity measured by these indices are thought to indicate a functional deprivation or localized functional disruption by the disease, whereas increased brain activity may reflect disease-related excessive function state in the specific brain region^29^.

Previous studies of ALFF/fALFF or ReHo have shown similar functional abnormalities in insomnia and schizophrenia. For instance, studies of insomnia patients reported that ALFF and ReHo changes had been observed in brain areas associated with emotion, cognition, and sleeping-wake system, such as the prefrontal cortex^30,31^, occipital lobe^30^, limbic system^32,33^ and cerebellum^30,31,33^, which were also been consistently found abnormal in patients with schizophrenia^34–36^. However, no experimental studies have directly compared the patterns of brain activity change between insomnia and schizophrenia and it remains unclear whether there exists a shared neurobiological pattern between insomnia and schizophrenia. An alternative strategy is to calculate the overlapping and specific mechanisms by comparing single-disorder effects^37,38^, although the levels of comorbidity between schizophrenia and insomnia may confound the effects.

Therefore, we conducted a coordinate-based analysis of likelihood estimate (ALE) meta-analysis comparing rs-fMRI studies of insomnia patients with those of schizophrenia. Given the limited literature on directly comparing SCZ and insomnia, we included studies that separately compared each patient group (insomnia or SCZ) to healthy populations and combined the single group activations to explore the conjunction and contrast areas between the two diseases. This is intended to enable a better understanding of the common and distinct pathophysiology of insomnia and schizophrenia and ultimately enlighten the adaption and improvement of treatment for schizophrenia patients with insomnia.

## Methods

### Search strategy

According to the Preferred Reporting Items for Systematic Reviews and Meta-Analyses (PRISMA) statement^39^, we collected relevant studies by a search of the PubMed, EMBASE and Web of Science databases up to December 2021. Keywords for the search were as follows: (1) [(“rest-state” OR “resting state”) AND (“functional magnetic resonance” OR “fMRI” OR “functional MRI”) AND (“insomnia” OR “insomnia disorder”)]; (2) [(“schizophrenia” OR “schizoaffective disorder” OR “schizophrenic disorder”) AND (“amplitude of low-frequency fluctuation” OR “ALFF” OR “low-frequency fluctuation” OR “ReHo” OR “regional homogeneity”)]. We also checked the reference lists of the included studies and relevant review articles to identify potential studies that were not included in the systematic search. This meta-analysis is registered in the PROSPERO database (ID: CRD42021287152).

### Study selection

A study was included in the meta-analysis if it satisfied the following criteria: (1) ALFF/fALFF or ReHo comparison of patients with schizophrenia/insomnia versus healthy controls(HC) was conducted; (2) whole‐brain coordinates in either Talairach or Montreal Neurological Institute (MNI) space can be acquired from the literature or Supplementary Materials; (3) The results were reported by using thresholds for significance that were corrected for multiple comparisons or uncorrected with spatial extent thresholds; (4) The study was written in English in the peer-reviewed journal.

The exclusion criteria were as follows: (1) case reports, conference abstracts, editorials, comments, or review studies without original data; (2) Studies using seed-voxel-based analysis or reporting only region-of-interest results; (3) The peak coordinates were not retrieved from the literature, even after contacting with the authors; (4) The number of each group in the study was <10; (5) studies of which data overlapped with those of other publications. In this case, we only included the study with the largest sample in our meta-analysis.

### Data extraction

Two authors (ZG and YX) independently conducted the literature search, study selection, and data extraction. All information was double-checked. If a disagreement appeared, a third author mediated for the final decision. The demographic and clinical data, imaging parameters, statistical information, and complete stereotaxic peak coordinates were extracted from each included study. The coordinates reported in Talairach were converted into MNI space by the icbm2tal transform in the GingerALE^40^.

Notably, A study refers to an individual publication and an experiment refers to the single specific contrast producing some peak coordinates. To decrease within-group effects^41^, we merged the results of one study into a maximum of two experiments (increases and decreases in brain activity, i.e., patients > control and patients < control) in the meta-analysis.

### Quality assessment

Each selected study was assessed for quality with a 12-point checklist which has been applied frequently in prior neuroimaging meta-analysis^42,43^. Two researchers (ZG and YX) independently evaluated the included studies from the following categories containing 12 items: participants (item 1–4), methods for image acquisition and analysis (item 5–10), and the results and conclusions (item 11,12). Each item received a score of 1, 0.5 or 0 based on the criteria that were fully, partially met, or not met, separately. More information of the checklist could be seen in Supplementary Materials.

### Activation likelihood estimation (ALE) meta-analysis

The current coordinate-based meta-analysis was conducted by the GingerALE software version 3.0.2 (http://brainmap.org/ale/), which implements the latest ALE algorithm^44^. Specifically speaking, ALE is an approach of meta-analysis that collects activation foci reported in neuroimaging studies and creates three-dimensional Gaussian probability distributions for each focus to compute modeled activation (MA) maps. ALE images are then obtained by the union of MA maps. Finally, a convergence of foci is verified by testing against the null-hypothesis of random spatial association between experiments^44,45^.

All coordinates extracted from the included studies were integrated and separated into four groups based on the regional brain activity changes of patients compared with HC: SCZ > HC, SCZ < HC, insomnia > HC, and insomnia < HC. A two-step analysis plan was utilized. Firstly, we conducted first-level ALE analyses respectively for the four contrasts, with an initial uncorrected threshold of voxel-level *p* < 0.005 and a minimum cluster size of 20 mm^3^. Then, the ALE results of these first-level analyses were pooled into the second-level conjunction/subtraction analysis to identify the common and distinct brain activation between insomnia and schizophrenia. Two second-level models were built to examine our concern: one comparing increased activities (i.e., insomnia > HC and SCZ > HC) and the other comparing decreased activities (i.e., insomnia < HC and SCZ < HC). The second-level analysis involved quantitative conjunction analysis and conducted non-parametric permutation simulations (10,000 permutations) to draw statistical inferences of differences between insomnia and SCZ. Because of the explorative nature of this study, we utilized an uncorrected threshold of *p* < 0.05 with a minimum cluster volume of 20 mm^3^ to remove false small-size clusters as well as discovering more co-activated areas. To visualize the final results, the ALE cluster maps were exported into Mango (http://www.nitrc.org/projects/mango), utilizing an anatomical brain template (Colin27_T1_seg_ MNI.nii).

### Subgroup analysis based on the phase of schizophrenia

The schizophrenia literature consists of both first-episode schizophrenia (FES) and long-term ill schizophrenia. Since growing evidence demonstrated that the patterns of structure and functional abnormalities may change during the course of schizophrenia^46–49^, whether illness course could exert an influence on the brain activation between insomnia and schizophrenia was worth investigating. To address this concern, we conducted an additional subgroup analysis of patients with first-episode and long-term ill schizophrenia. The classification of the phase of schizophrenia was based on criteria defined by the authors of the original study. Studies including both first-episode and long-term ill schizophrenia were excluded from the subgroup analysis.

### Control analyses

To evaluate the robustness of our conjunction results and control the age-related effects, we conducted an additional control analysis. First, we excluded studies of children and adolescents and repeated conjunction and contrast analysis only for studies involving adults. Second, since the participants in insomnia studies were older than those in SCZ studies, we ran the same analysis but only included the schizophrenia literature solely to those studies involving participants whose ages are above 30 to narrow the age gap between participants of insomnia and SCZ.

## Results

### Study selection and characteristics

**Fig. 1:**
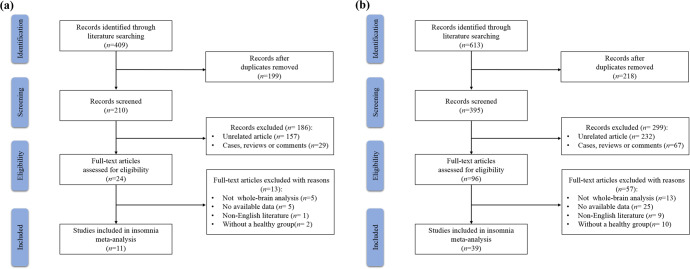
Flow diagram for the inclusion and exclusion of studies. Flow diagram of insomnia meta-analysis study selection (**a**) and SCZ meta-analysis study selection (**b**). SCZ schizophrenia.

Figure 1 shows the flow diagram which depicts the process of reviewing and selecting included studies. Ultimately, we included 11 studies of insomnia and 39 studies of schizophrenia, respectively. The final datasets comprised 360 insomnia patients (mean age 43.1 years), 1945 SCZ patients (mean age 28.5 years), and 2216 HC (372 from insomnia vs. HC studies, mean age 41.6 years; and 1844 from SCZ vs. HC studies, mean age 29.1 years). The literature researching insomnia yield 60 foci of brain activation and the studies about SCZ provide 342 significant foci. For all included studies, 10 experiments reported HC > insomnia,10 for insomnia > HC, whereas 36 experiments reported HC > SCZ and 32 experiments for SCZ > HC. The detailed demographic, clinical and imaging characteristics of the included studies are summarized in Table 1.

**Table 1:** Characteristics of the included studies of insomnia (A) and SCZ (B) in the meta-analysis.

Study (year)	Control		Patients		Duration of illness (year)	PSQI		Methodology	Scanner	FWHM (mm)	Threshold and correction method
	Numbers/ males	Age (mean ± SD)	Numbers/ males	Age (mean ± SD)		Control	Patients				
**A**											
Dai et al.^104^	42/18	49.1 ± 10.2	42/15	49.2 ± 11.0	5.4 ± 5.2	2.5 ± 0.9	15.2 ± 2.2	ALFF	3.0 T Siemens	6	AlphaSim *p* < 0.05
Li et al.^30^	44/11	39.9 ± 9.4	55/24	39.2 ± 10.3	3.8 ± 2.5	5.9 ± 2.3	12.5 ± 3.3	ALFF	1.5 T Philips	6	AlphaSim *p* < 0.05
Liu et al.^105^	71/33	36.1 ± 12.5	31/16	38.6 ± 12.1	NA	NA	NA	fALFF	3.0 T Siemens	4	AlphaSim *p* < 0.05
Ran et al.^106^	20/6	38.7 ± 7.4	21/6	40.6 ± 7.5	NA	2.9 ± 1.0	13.3 ± 3.0	ALFF	3.0 T GE	6	AlphaSim *p* < 0.05
Wang et al.^107^	15/7	45.5 ± 12.7	30/12	49.7 ± 9.6	NA	3.5 ± 0.9	15.2 ± 1.7	fALFF	3.0 T Siemens	6	FWE correct *p* < 0.05
Zhao et al.^108^	20/8	36.2 ± 0.3	22/9	42.6 ± 14.5	NA	4.6 ± 1.4	12.4 ± 3.5	ALFF	3.0 T Siemens	6	Alphasim *p* < 0.05
Zhou et al.^31^	27/17	40.9 ± 11.5	27/17	42.6 ± 11.6	11.0 ± 8.9	0.9 ± 1.1	13.3 ± 2.5	ALFF	3.0 T Siemens	6	Alphasim *p* < 0.05
Dai et al.^33^	24/12	52.5 ± 6.6	24/7	54.8 ± 9.8	6.0 ± 5.2	2.3 ± 0.8	15.6 ± 2.1	ReHo	3.0 T Siemens	6	Alphasim *p* < 0.05
Pang et al.^109^	28/5	51.3 ± 12.5	17/3	49.0 ± 14.3	NA	0.4 ± 0.6	12.6 ± 3.1	ReHo	1.5 T Philips	NA	Uncorrected *p* < 0.05
Wang et al.^32^	47/14	40.0 ± 9.1	59/21	39.3 ± 10.7	NA	5.8 ± 2.3	12.4 ± 3.3	ReHo	1.5 T Philips	8	AlphaSim *p* < 0.05
Zhang et al.^110^	34/13	35.8 ± 7.1	32/12	37.5 ± 8.9	NA	2.1 ± 1.4	12.0 ± 3.6	ReHo	3.0 T Siemens	6	AlphaSim *p* < 0.05

Study (year)	Control		Patients		Duration of illness (month)	PANSS				State	Methodology	Scanner	FWHM (mm)	Threshold and correction method
	Numbers /males	Age (mean ± SD)	Numbers/males	Age (mean ± SD)		Total score	Positive score	Negative score	General score					
**B**														
Alonso-Solis et al.^111^^a^	20/13	37.8 ± 7.4	19/13 14/8	40.1 ± 8.9 36.4 ± 7.1	193.3 ± 111.6 96.0 ± 74.4	NA	17.9 ± 4.9 11.4 ± 4.3	21.5 ± 5.9 14.4 ± 4.7	34.2 ± 7.9 27.4 ± 5.7	long term ill SCZ	ALFF/fALFF	3.0 T Philips	6	Corrected *p* < 0.05
Bai et al.^112^	17/14	28.7 ± 6.0	17/14	26.0 ± 5.5	40.3 ± 38.0	82.1 ± 12.3	15.3 ± 5.5	24.9 ± 6.5	41.9 ± 7.3	FES and long term ill SCZ	ReHo	1.5 T GE	4	AlphaSim *p* < 0.05
Cui et al.^113a^	19/10	23.8 ± 3.8	17/10 15/8	21.2 ± 3.9 22.5 ± 4.1	6.5 ± 6.0 10.2 ± 18.2	106.2 ± 13.9 88.1 ± 26.2	31.1 ± 7.1 17.9 ± 9.3	25.5 ± 3.8 22.7 ± 10.7	49.6 ± 9.3 47.4 ± 10.4	FES	ReHo and ALFF	3.0 T Siemens	4	AlphaSim *p* < 0.01
Fryer et al.^114^	86/51	22.7 ± 6.5	74/49	21.9 ± 4.2	NA	NA	13.9 ± 4.8	17.3 ± 6.6	33.3 ± 8.7	FES	fALFF	3.0 T Siemens	6	FWE correction *p* < 0.05
Gao et al.^115^	14/9	34.9 ± 13.6	14/9	33.2 ± 10.7	110.4 ± ‘102.0	74.1 ± 16.2	16.4 ± 5.3	22.6 ± 6.2	30.8 ± 8.7	long term ill SCZ	ReHo	1.5 T Siemens	6	AlphaSim *p* < 0.05
Gao et al.^116a^	29/16	32.7 ± 7.6	17/10 17/9	31.2 ± 9.4 36.8 ± 9.1	168.0 ± 105.0 94.6 ± 56.6	97.8 ± 11.1 37.3 ± 6.8	27.5 ± 6.0 9.5 ± 3.1	21.1 ± 3.9 8.4 ± 1.9	49.1 ± 5.5 19.2 ± 2.6	long term ill SCZ	ReHo	3.0 T Siemens	4	GRF correction *p* < 0.05
Gao et al.^117^	50/23	28.4 ± 6.9	57/20	31.6 ± 11.4	30.2 ± 32.6	91.8 ± 14.2	26.4 ± 4.9	20.7 ± 6.9	44.8 ± 7.4	FES	ReHo	3.0 T Siemens	4	GRF correction *p* < 0.05
Gou et al.^118^	21/14	28.8 ± 6.1	28/16	23.9 ± 5.4	15.1 ± 14.2	85.7 ± 19.9	17.8 ± 6.5	21.0 ± 6.2	38.9 ± 9.4	FES and long term ill SCZ	ReHo	1.5 T GE	4	AlphaSim *p* < 0.005
He et al.^95^	104/49	26.6 ± 8.9	104/50	25.4 ± 8.3	9.9 ± 8.0	92.1 ± 17.5	NA	NA	NA	FES	fALFF	3.0 T GE	6	FDR *p* < 0.05
Hoptman et al.^58^	26/19	41.9 ± 10.9	29/26	36.5 ± 11.0	156.0 ± 86.4	76.5 ± 16.6	18.4 ± 6.2	20.2 ± 6.2	NA	long term ill SCZ	ALFF,fALFF	1.5 T Siemens	6	Uncorrected *p* < 0.05
Hu et al.^90^	38/25	24.8 ± 4.6	42/27	24.9 ± 4.8	8.4 ± 2.6	91.9 ± 11.2	25.6 ± 3.8	18.2 ± 5.2	48.1 ± 6.5	FES	fALFF,ReHo	3.0 T Siemens	8	AlphaSim *p* < 0.05
Huang et al.^96^	66/30	24.5 ± 8.6	66/30	24.2 ± 8.4	8.8 ± 14.1	107.2 ± 15.1	26.4 ± 5.2	20.7 ± 6.3	51.3 ± 9.2	FES	ALFF	3.0 T GE	8	Corrected *p* < 0.05
Jin et al.^119^	24/12	30.9 ± 6.3	23/11	31.74 ± 6.71	NA	NA	NA	NA	NA	FES	ReHo	3.0 T Philips	6	FDR *p* < 0.05
Lei et al. 2015^120^	102/50	24.8 ± 6.9	124/61	24.5 ± 6.7	6.9 ± 8.5	88.4 ± 17.1	15.3 ± 4.3	15.7 ± 8.1	NA	FES	ALFF	3.0 T GE	6	AlphaSim *p* < 0.05
Li et al.^121^	16/7	22.4 ± 4.4	20/6	22.9 ± 8.5	6.4 ± 13.6	101.6 ± 12.3	25.1 ± 5.7	18.8 ± 7.4	49.7 ± 7.5	FES	ALFF	3.0 T GE	8	AlphaSim *p* < 0.05
Li et al.^122a^	42/24	23.3 ± 7.3	41/23 42/25	23.3 ± 6.9 22.9 ± 6.7	20.2 ± 14.0 19.8 ± 13.0	86.2 ± 15.7 85.8 ± 12.8	25.4 ± 6.2 21.6 ± 4.9	16.5 ± 5.9 23.2 ± 5.8	43.4 ± 9.5 41.3 ± 6.4	FES	ALFF	3.0 T GE	6	AlphaSim *p* < 0.05
Lian et al.^123^	30/16	20.5 ± 2.1	18/8	20.4 ± 3.0	7.9 ± 2.9	80.5 ± 11.4	19.1 ± 4.5	21.5 ± 5.5	NA	FES	fALFF	3.0 T Philip	NA	FDR *p* < 0.01
Liu et al.^124^	27/18	27.4 ± 7.2	27/15	25.4 ± 5.9	18.3 ± 15.8	85.8 ± 12.8	21.6 ± 4.9	23.2 ± 5.8	41.3 ± 6.4	FES	ALFF,ReHo	1.5 T GE	8	FWE correction *p* < 0.05
Liu et al.^125^	18/9	24.4 ± 3.9	18/9	23.7 ± 4.4	26.8 ± 19.2	80.4 ± 18.7	NA	NA	NA	long term ill SCZ	ReHo	1.5 T GE	4	AlphaSim *p* < 0.05
Liu et al.^97a^	21/14	31.4 ± 8.9	21/19 21/15	29.1 ± 10.0 31.0 ± 9.8	85.2 ± 105.1 56.9 ± 41.3	68.1 ± 26.2 60.9 ± 20.0	14.1 ± 7.5 11.6 ± 7.0	19.5 ± 7.9 22.2 ± 9.0	34.5 ± 14.1 27.1 ± 9.9	FES and long term ill SCZ	ALFF	3.0 T GE	8	AlphaSim *p* < 0.05
Liu et al.^126^	31/14	15.4 ± 1.5	48/21	15.8 ± 1.6	5.4 ± 6.1	75.1 ± 9.9	21.5 ± 5.0	17.9 ± 7.0	34.3 ± 5.9	FES	ReHo	3.0 T Siemens	NA	Corrected *p* < 0.05
Lui et al. 2010^89^	34/13	25 ± 8.0	34/13	24.6 ± 8.5	7.8 ± 12.4	104.2 ± 13.9	26.9 ± 5.6	19.1 ± 6.2	49.9 ± 8.1	FES	ALFF	3.0 T GE	8	FWE correction *p* < 0.05
Lui et al.^35^	59/26	38.0 ± 17.0	37/22	36.0 ± 14.0	177.7 ± 143.9	71.2 ± 15.5	18.2 ± 4.8	18.3 ± 6.5	34.7 ± 7.4	long term ill SCZ	ALFF	3.0 T GE	8	AlphaSim *p* < 0.05
Ren et al.^36^	100/41	24.4 ± 7.6	100/41	24.3 ± 7.5	6.3 ± 11.0	97.9 ± 17.8	25.1 ± 6.0	18.8 ± 7.7	47.6 ± 9.6	FES	ALFF	3.0 T GE	8	AlphaSim *p* < 0.05
Salvador et al.^127^	122/82	36.5 ± 10.7	116/81	36.8 ± 11.1	178.6 ± 143.4	69.2 ± 18.6	16.6 ± 5.6	19.6 ± 6.8	33.1 ± 9.2	long term ill SCZ	ALFF	1.5 T GE	NA	FWE correction *p* < 0.05
Shan et al.^128a^	20/14	25.7 ± 4.9	19/12 20/15	26.1 ± 5.8 22.8 ± 4.4	NA	104.8 ± 10.0 103.0 ± 10.8	24.9 ± 3.1 22.8 ± 5.8	27.2 ± 5.2 27.4 ± 5.4	52.8 ± 5.2 52.8 ± 5.1	long term ill SCZ	ReHo	3.0 T Siemens	4	FDR correction *p* < 0.05
Tang et al.^129^	59/27	20.9 ± 4.0	42/21	19.0 ± 4.0	6.6 ± 10.6	NA	NA	NA	NA	FES	ALFF	3.0 T GE	6	Corrected *p* < 0.01
Turner et al.^61^	160/114	37.0 ± 10.4	146/111	38.0 ± 11.3	205.1 (24–456)	57.6 (37–88)	15.0 (8–25)	14.4 (7–29)	28.1 (18–43)	long term ill SCZ	ALFF/fALFF	3.0 T Siemens	8	FDR correction *p* < 0.05
Wang et al.^130^	60/23	30.1 ± 8.5	44/13	25.0 ± 7.5	43.0 ± 42.4	NA	NA	NA	NA	FES and Long term ill SCZ	ALFF	3.0 T GE	6	GRF correction *p* < 0.001
Wu et al.^131^	32/21	31.4 ± 7.8	32/16	30.9 ± 8.3	8.9 ± 6.4	77.4 ± 5.2	20.0 ± 4.3	20.6 ± 3.5	36.8 ± 3.7	FES	fALFF	3.0 T Siemens	NA	GRF correction *p* < 0.05
Xie et al.^132^	33/13	32.0 ± 7.3	30/13	30.3 ± 4.5	21.4 ± 4.9	79.9 ± 10.6	19.7 ± 4.6	NA	40.4 ± 6.7	long term ill SCZ	ALFF	3.0 T GE	6	FDR correction *p* < 0.05
Yan et al.^54^	74/45	26.3 ± 7.0	69/50	24.2 ± 7.1	13.7 ± 11.8	84.2 ± 8.3	24.4 ± 3.9	17.6 ± 4.1	42.2 ± 3.6	FES	ReHo	3.0 T Siemens	4	FWE correction *p* < 0.05
Yang et al.^133^	39/9	40.9 ± 6.3	37/9	39.7 ± 10.9	204.0 ± 105.4	76.0 ± 18.5	13.6 ± 5.4	23.9 ± 7.6	NA	long term ill SCZ	ReHo	3.0 T Phillips	4	AlphaSim *p* < 0.05
Yang et al.^134^	93/46	30.1 ± 6.8	64/28	28.6 ± 4.4	37.4 ± 38.9	80.8 ± 8.9	NA	NA	NA	FES and Long term ill SCZ	fALFF	3.0 T Siemens	6	uncorrected *p* < 0.05
Yu et al.^59,60^	62/NA	29.9 ± 8.6	69/NA	31.7 ± 9.6	85.2 ± 78.0	52.9 ± 16.8	12.1 ± 4.7	13.4 ± 6.1	27.4 ± 9.6	long term ill SCZ	ALFF/fALFF, ReHo	3.0 T Siemens	NA	FWE correction *p* < 0.05
Zhao et al.^135^	26/17	22.6 ± 3.7	44/31	23.7 ± 5.3	12.0 ± 9.2	102.0 ± 16.7	15.3 ± 3.3	24.7 ± 7.5	NA	FES	ReHo	3.0 T Siemens	4	TFCE correction *p* < 0.01
Zheng et al.^136^	30/13	15.4 ± 1.5	35/20	15.5 ± 1.8	6.6 ± 6.7	74.6 ± 10.6	20.4 ± 5.7	20.9 ± 8.4	33.3 ± 6.7	FES	ALFF	3.0 T Siemens	6	AlphaSim *p* < 0.05
Zhou et al.^137a^	40/40	46.0 ± 9.5	33/33 41/41	48.7 ± 7.7 46.0 ± 5.4	319.3 ± 84.7 282.1 ± 69.7	NA	NA	NA	NA	long term ill SCZ	fALFF	3.0 T GE	4	Corrected *p* < 0.05

*FWE* family-wise error, *GRF* Gaussian random field, *FDR* false discovery rate, *FWHM* full width at half maximum, *NA* not available, *PSQI* Pittsburgh Sleep Quality Index, *PANSS* Positive and Negative Syndrome Scale, *SD* standard deviation, *SCZ* schizophrenia.

^a^Two datasets are included.

The qualities of all included studies were considered to be similar and high. The mean quality scores of included studies for insomnia and SCZ were 11.8 (range 11.5–12) and 11.8 (range 11–12) respectively. The detailed quality scores of each included study are showed in Table S1.

### Results of the main meta-analysis

Results from the first-level ALE analyses of insomnia and SCZ studies are presented in Supplementary Tables S2–S5. The conjunction and contrast analyses of insomnia and SCZ literature provide the clusters of overlapping increased and decreased brain activities, as well as the significant different brain activations between the two diseases. The second-level results are discussed below in detail.

**Fig. 2:**
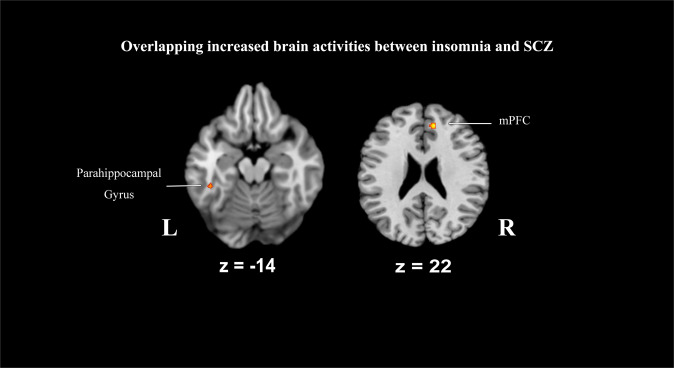
Pattern of overlapping increased activities between insomnia and SCZ. SCZ schizophrenia, L left, R right. Conjunction analysis (*p* < 0.05, *k* = 20) identified two clusters of increased brain activity in both insomnia and SCZ patients: the right medial prefrontal gyrus (mPFC, *k* = 136, ALE value = 0.009) and the left parahippocampal gyrus (*k* = 56, ALE value = 0.007).

The conjunction analysis demonstrates the common region with increased activity in frontolimbic structures, including the right medial prefrontal gyrus (mPFC) and left parahippocampal gyrus (Fig. 2, Table 2A). We found no significant direct overlapping area in decreased activities between insomnia and SCZ likewise.

**Table 2:** Clusters demonstrating the overlapping and distinct brain activation regions between insomnia and SCZ.

Volume (mm^3^)	BA	Hemisphere	Label	MNI coordinate		
				x	y	z
**A. Overlapping increased activities (insomnia** > **HC and SCZ** > **HC)**						
136	9	R	Medial Prerontal Gyrus (mPFC)	12	42	22
56	36	L	Parahippocampal Gyrus	−42	−38	−12
**B. Significantly greater increased activities in insomnia vs SCZ**						
40	23	R	Posterior Cingulate	9.3	−54.7	18
**C. Significantly greater increased activities in SCZ vs insomnia**						
2480		R	Caudate Body, Putmen	26	12	8
				14.9	9.3	12.2
				15.4	9	6.9
				20	6	14
				22	6	10
				20	4	6
				9	13	8
				16	−0.8	18
840		R	Putamen, Caudate Head	23	14	−2
				20	18	−4
				24	18	0
				28	16	−8
808		L	Putamen, Caudate Body, Caudate Head, Lateral Globus Pallidus	−10	10	4
				−20	12	2
				−13	8	−1
				−17	9	2
				−4	12	6
656		L	Putamen, Caudate Body	−22	−2	16
				−22	2	12
				−18	0	12
				−24	−2	12
				−22	−6	12
				−16	4	18
616	13	R	Insula	43.4	18.1	8.9
				49.1	17.1	12.3
				40	22	12.7
80		R	Putamen	32	4	10
80	13	R	Insula	36	29	7
40	44	R	Precentral Gyrus	48	10	4
24		L	Caudate Body	−2	14.7	5.3
24	13	R	Insula	37.3	27.3	14
**D. Significantly greater decreased activities in insomnia vs SCZ**						
104	13	R	Insula	47.8	5.1	−2.9
96		R	Cerebellum, Inferior Semi-Lunar Lobule	7.7	−68.5	−46.5
56	25	R	Inferior Frontal Gyrus	53.1	18	−10.9
48		R	Medial Frontal Gyrus	14.7	15.7	−26.3
32		R	Cerebellar Tonsil	12.5	−62	−47.5
24		L	Cingulate Gyrus	0	−40	36
**E. Significantly greater decreased activities in SCZ vs insomnia**						
344	4	R	Precentral Gyrus	52.6	−12.7	44.7
184	11	L	Orbital Frontal Cortex (OFC)	−7.3	52	−23.3

*BA* Brodmann area, *MNI* Montreal Neurological Institute, *SCZ* schizophrenia, *HC* healthy control, *R* right, *L* left.

**Fig. 3:**
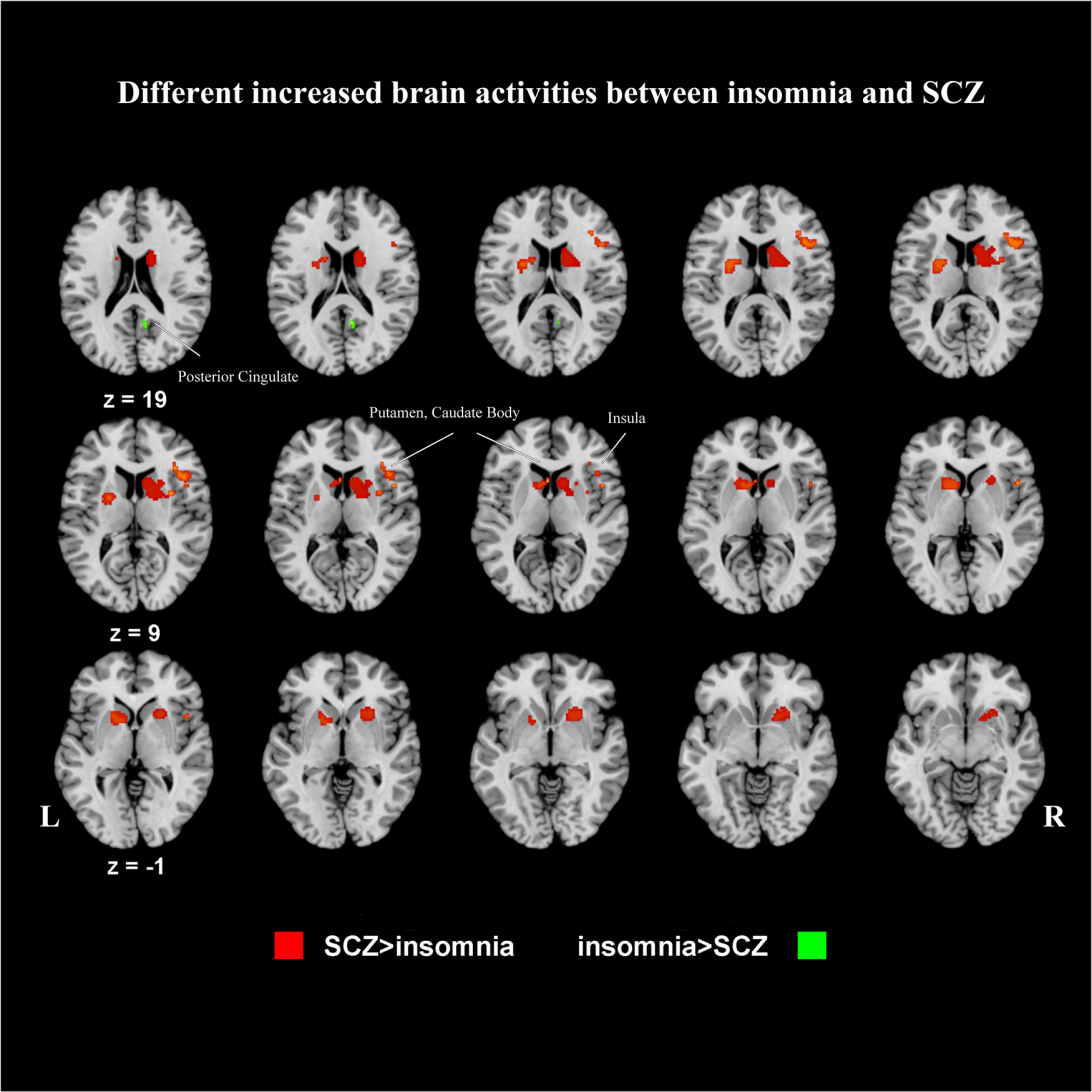
Patterns of different increased activities between insomnia and SCZ. SCZ schizophrenia, L left, R right. Contrast analysis shows areas with increased activities which were significantly different between insomnia and SCZ patients. Clusters with greater increased activities in SCZ are shown in red, and clusters with greater increased activities in insomnia are shown in green. First-level analyses were formed using a threshold of *p* < 0.005, *k* = 20 mm^3^, and second analyses were conducted with a threshold of *p* < 0.05, *k* = 20 mm, 10,000 permutations.

**Fig. 4:**
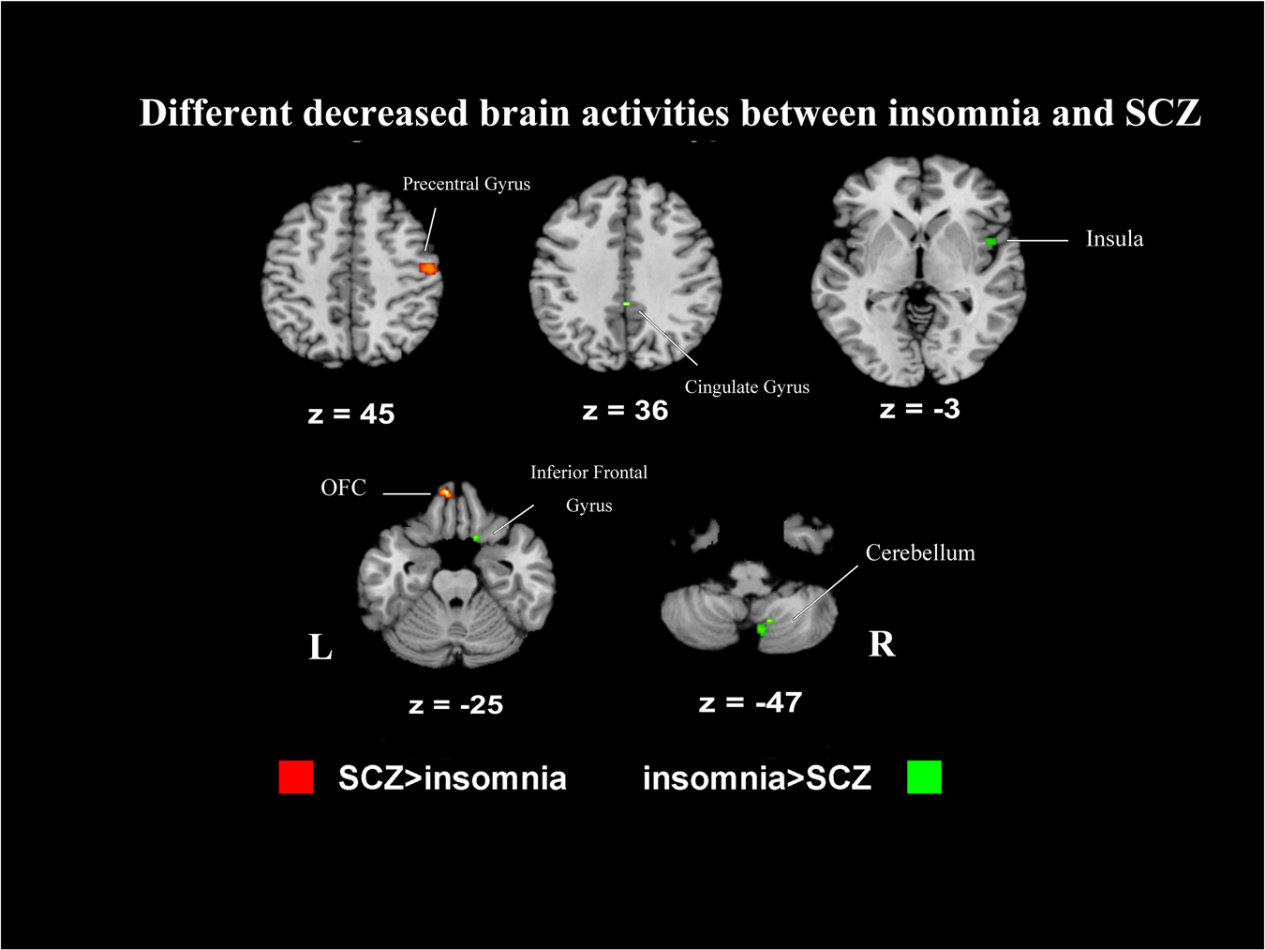
Patterns of different decreased activities between insomnia and SCZ. *SCZ* schizophrenia, *L* left, R right. Contrast analysis shows areas with decreased activities which were significantly different between insomnia and SCZ patients. Clusters with greater decreased activities in SCZ are shown in red, and clusters with greater decreased activities in insomnia are shown in green. First-level analyses were formed using a threshold of *p* < 0.005, *k* = 20 mm^3^, and second analyses were conducted with a threshold of *p* < 0.05, *k* = 20 mm, 10,000 permutations.

Subtractions of first-level analyses revealed distinct patterns of activation between insomnia and SCZ. Compared with those with SCZ, patients with insomnia exhibited greater increased activities in right posterior cingulate (Fig. 3, Table 2B) and greater decreased activities in right insula, frontal lobe, cerebellum and left cingulate gyrus (Fig. 4, Table 2D).

In contrast, the analyses found greater increased activities in the subcortical area, including bilateral putamen and caudate body, right insula (Fig. 3, Table 2C), as well as greater decreased activities in the right precentral gyrus and left orbitofrontal gyrus (Fig. 4, Table 2E) in SCZ relative to insomnia.

### Results of subgroup analysis

To estimate the effect of illness duration on the main results, we repeated the conjunction and contrast analysis for FES and long-term ill SCZ, respectively. In the FES subgroup, the distinct patterns of regional brain activities between insomnia and FES are similar to those in the main results. In the long-term ill SCZ subgroup, we found overlapping increased activities in medial prefrontal gyrus and parahippocampus remain significant, and patients with long-term ill SCZ demonstrate greater decreased activities in right paracentral lobule, precuneus and left occipital gyrus. The detailed results can be found in Table S6.

### Control analysis

After excluding two adolescent schizophrenia studies, the main results with only adult participants remain largely unchanged. The detailed results were provided in Table S7. When the analyses were restricted to studies with participants with older age (15 experiments in HC > SCZ contrast; 15 experiments in SCZ > HC contrast), the common hyperactivation in parahippocampal gyrus remained significant, although the increased activity in mPFC were not replicated in the relatively low-power analysis. Moreover, the greater increased activities in subcortical areas in SCZ and decreased activities in precentral gyrus were also replicated in the subgroup analysis (Table S8).

## Discussion

In the current study, we conducted a coordinate-based meta-analysis that investigates the common and distinct patterns of brain activity between insomnia and SCZ by comparing patients with healthy controls and contrasting the activation areas in insomnia and SCZ patients. The results displayed a convergence of increased activities within frontolimbic structures containing right mPFC and left parahippocampal gyrus. Moreover, the overlapping increased brain activities were accompanied by specific distinct increased activity within subcortical areas and decreased activativity within precentral gyrus and ventromedial prefrontal gyrus (VMPFC) in SCZ. These results support a hypothesis that explains the associations between SCZ and insomnia: the common abnormality in areas of attention, vigilance and emotion control and common impairments in areas of memory and cognition, but probably different mechanisms of sleep disturbance in SCZ compared with primary insomnia. Although the unknown comorbidity between the two diseases may limit our conclusion, our findings can offer novel insights into the complicated interplay between insomnia and schizophrenia to further explore the potential treatment target for schizophrenia patients with insomnia symptoms.

Our meta-analysis demonstrated a convergent increased activation pattern within right mPFC. mPFC is a crucial region for integrating input information and delivering integrated information to output structures^50^. It has been proved to play an important role in emotion control, attention and vigilance^51–53^, in which deficits have been shown in SCZ patients^31,54,55^. Interestingly, mPFC is also found to generate non-rapid eye movement (NREM) slow-wave oscillations and as a result, to participate in mediating the induction and maintenance of different sleep stage^56,57^. In addition, abnormal increased brain activities in the mPFC have been reported in both SCZ^54,58–61^ and insomnia patients^31,55,62^previously. Since mPFC is a key node for default network (DMN), it is likely that the dysregulation of DMN plays a pivotal role in the common pathophysiology of insomnia and SCZ. The increased activities of mPFC may disrupt the decoupling of DMN during sleep^63^ and lead to aberrant thalamocortical sleep-wakefulness rhythms^64,65^. Similarly, the failure of deactivation in DMN could be observed in SCZ patients^66^ and correlated to dysfunctional connectivity to thalamus^67^. This can enlighten future studies on exploring the main circuit responsible for insomnia symptoms in SCZ.

Besides neuroimaging studies, genetic studies have indicated mPFC as a potential key region in the pathophysiological mechanisms behind insomnia problems in SCZ. Shen et al. found that deleting FXR1 gene, of which the variants are linked to shorter sleep duration^68^, from parvalbumin interneurons of mPFC leads to impaired mPFC gamma oscillation and SCZ-like behaviors^69^. The large GWAS study in 1,331,010 individuals also showed insomnia-related gene set enrichments in frontal cortex, which have correlations with psychiatric traits^70^. Considering together with those findings, our results consolidate the hypothesis that mPFC is a key neuroanatomic structure that is responsible for insomnia symptom in SCZ patients.

In addition to the hyperactivation in mPFC, our study also revealed the convergent increased activity in the parahippocampal gyrus. Increased activity in the parahippocampal region has been consistently reported by previous meta-analyses on functional activation patterns in both SCZ and insomnia^71,72^. As an important component of the limbic system, hippo/parahippocampal gyrus has been demonstrated to be associated with lots of cognitive processes, including semantic processing, visuospatial navigation, working memory and episodic memory^73–76^. Our results are in agreement with the theory that the aberrant activations of parahippocampal gyrus are correlated with impaired cognition and memory, which has been found in both SCZ^35,77,78^ and insomnia patients^79^. Moreover, the medial prefrontal cortex and parahippocampal gyrus are interconnected by cingulum bundle structurally^80^ and belong to DMN functionally. Our results revealed that the abnormal activation of DMN was presumed to be the common target for the neurobiological mechanisms of SCZ and insomnia, which was found to be altered in the two diseases separately^81,82^.

Apart from the functional convergent pattern between SCZ and insomnia patients, our study also demonstrated the main greater increased activities in the subcortical regions in SCZ compared to insomnia. Increased activities in subcortical regions including insula, striatum, and caudate body have been extensively reported by resting-state fMRI-based meta-analyses in SCZ^49,83^. Broad evidence from fMRI studies has revealed that the subcortical areas containing insula extending into the striatal regions are implicated in autonomic, affective, cognitive and behavioral processing^84–87^, deficits of which are the core manifestations of SCZ. Previous studies have also unraveled the association between activation of subcortical regions and impairment of cognitive-emotional control in SCZ^88^. Another possibility is that antipsychotic medication could also increase activities in basal ganglia region^89,90^. The increased activities of subcortical regions may cause high emotional vulnerability^84^ and other psychiatric symptoms^91^, as well as dysregulation of autonomic control, which will regulate cardiovascular and neural activities during sleep and waking^92^. These abnormalities constitute the “sleep stressor” and lead the brain into a hyper-arousal state, preventing the SCZ patients from sound sleep. This hypothesis could be supported by the findings that sedating atypical antipsychotics like olanzapine and paliperidone may ameliorate sleep parameters in SCZ patients^93,94^. Thus, more studies targeting the neurobiological function of subcortical regions in SCZ comorbid insomnia are warranted to be conducted.

As for decreased brain activities, our results showed the different patterns between SCZ and insomnia patients. In SCZ patients, precentral gyrus and orbitofrontal gyrus were found to be deactivated to a higher degree. Previous fMRI studies have found decreased ReHo/ALFF values in the OFC and precentral gyrus in either first-episode or long-term ill schizophrenia^35,95–97^. OFC and precentral gyrus are both participated in sensorimotor integration and their hypoactivations are correlated with negative symptoms including motor retardation and blunted affection in SCZ^98,99^, which are commonly not appeared in insomnia. Conversely, the decreased activation regions in insomnia patients are distributed more broadly. These regions including cerebellum, insula, cingulate gyrus and frontal gyrus were found to play a role in sleep disorders. Their morphological and functional changes indicated abnormal neural activity and may be correlated with disturbance of neural electrical activities in REM and NREM sleep^57,100–102^. More studies combined with EEG and neuroimaging techniques are required to investigate how these regions regulate the sleep-wake circle and cause sleep disturbance in the future.

Furthermore, our subgroup analysis found the effect of illness duration in SCZ on our results. Most results in the main analysis were still robust even in consideration of illness duration. The distinct patterns of brain activation in FES and long-term illness SCZ are also consistent with those reported in the previous meta-analyses^49,103^. The activation difference between different phases of SCZ may reflect the progressive impairment and change of brain function along the illness course. However, the effects of medication and lifestyle are still difficult to control and the clinical sleep-disturbance patterns in FES and long-term ill SCZ remains unclear, so more clinical and neuroimaging studies should be conducted to unravel the problem.

Several limitations should be considered when interpreting the findings in our meta-analysis. First, the proportion of schizophrenia patients with comorbid insomnia in the included studies is unavailable, which limited the explanation of the shared neural activity pattern of the two diseases. Further investigations directly focused on the comorbidity of SCZ and insomnia are required to confirm our findings. Second, for the explorative nature of this study, we adopted a relatively loose statistical threshold, which might increase false positive probability of the results. Third, our meta-analysis is based on the peak coordinates from the selected literature rather than on raw data, which includes nonsignificant results, which may limit the accuracy of our results. Fourth, on account of the limitation in the amount of literature, the studies on insomnia included in this meta-analysis are relatively less compared with the fMRI studies of SCZ. The difference in datasets of the two diseases may cause concerns of differential statistical power in the first-level analysis. Lastly, some factors including sex, age, and severity of the disease can impact brain activation. Considering our intention to identify the common and distinct pattern of brain activation between SCZ and insomnia by separately comparing the patients with healthy controls who matched the factors mentioned before, the effect of those factors between the SCZ studies and insomnia studies is limited.

## Conclusion

Despite those limitations, our current coordinate-based meta-analysis, drawing upon a large number of studies from both SCZ and insomnia literature, offers some novel findings on how the two diseases converge and differ from each other in the brain activation area. We identify the convergent increased brain activity pattern including mPFC and parahippocampal gyrus, providing a possible common pathway of SCZ with comorbid insomnia. Furthermore, we demonstrate the distinct activation patterns between the two diseases, most prominently including the increased activities of subcortical regions in SCZ, indicating the specific mechanisms of sleep disturbance in SCZ. In the future, more experiments are required to verify our conclusions and explore the potential value of these neurobiological patterns in the treatment of sleep problems in SCZ.

## Supplementary information

Supplementary Material
