# Supplementary material for: Comparisons of resting-state brain activity between insomnia and schizophrenia: a coordinate-based meta-analysis
Source: Schizophrenia (Heidelb). 2022 Oct 7;8(1):80. doi: 10.1038/s41537-022-00291-3 (PMC9547062; doi:10.1038/s41537-022-00291-3)
Supplement: Supplementary file 1 — Supplementary Material [file 41537_2022_291_MOESM1_ESM.docx]

Supplementary Material

**Table S1 Study quality assessment**

| Study(year) | Imaging Methodology Quality Assessment Checklist | | | | | | | | | | | | | | | | | | | | | | | | |  |  |
| --- | --- | --- | --- | --- | --- | --- | --- | --- | --- | --- | --- | --- | --- | --- | --- | --- | --- | --- | --- | --- | --- | --- | --- | --- | --- | --- | --- |
|  | 1 | | 2 | 3 | | 4 | | 5 | | 6 | | 7 | | 8 | | 9 | | 10 | | 11 | | 12 | | Overall | | |  |
| **Study of insomnia** | | | | | | | | | | | | | | | | | | | | | | | | | |  |  |
| Dai et al. 2016[100] | 1 | 1 | | | 1 | | 1 | | 1 | | 1 | | 1 | | 1 | | 1 | | 1 | | 1 | | 1 | | 12 | | |
| Li et al. 2016[29] | 1 | 1 | | | 0.5 | | 1 | | 1 | | 1 | | 1 | | 1 | | 1 | | 1 | | 1 | | 1 | | 11.5 | | |
| Liu et al. 2016[101] | 1 | 1 | | | 0.5 | | 1 | | 1 | | 1 | | 1 | | 1 | | 1 | | 1 | | 1 | | 1 | | 11.5 | | |
| Ran et al. 2017[102] | 1 | 0.5 | | | 1 | | 1 | | 1 | | 1 | | 1 | | 1 | | 1 | | 1 | | 1 | | 1 | | 11.5 | | |
| Wang et al. 2020[103] | 1 | 1 | | | 1 | | 1 | | 1 | | 1 | | 1 | | 1 | | 1 | | 1 | | 1 | | 1 | | 12 | | |
| Zhao et al. 2020[104] | 1 | 0.5 | | | 1 | | 1 | | 1 | | 1 | | 1 | | 1 | | 1 | | 1 | | 1 | | 1 | | 11.5 | | |
| Zhou et al. 2017[30] | 1 | 1 | | | 1 | | 1 | | 1 | | 1 | | 1 | | 1 | | 1 | | 1 | | 1 | | 1 | | 12 | | |
| Dai et al. 2014[32] | 1 | 1 | | | 1 | | 1 | | 1 | | 1 | | 1 | | 1 | | 1 | | 1 | | 1 | | 1 | | 12 | | |
| Pang et al. 2018[105] | 1 | 1 | | | 1 | | 1 | | 1 | | 1 | | 1 | | 1 | | 1 | | 1 | | 0.5 | | 1 | | 11.5 | | |
| Wang et al. 2016[31] | 1 | 1 | | | 1 | | 1 | | 1 | | 1 | | 1 | | 1 | | 1 | | 1 | | 1 | | 1 | | 12 | | |
| Zhang et al. 2021[106] | 1 | 1 | | | 1 | | 1 | | 1 | | 1 | | 1 | | 1 | | 1 | | 1 | | 1 | | 1 | | 12 | | |
| **Study of SCZ** | | | | | | | | | | | | | | | | | | | | | | | | | |  |  |
| Alonso-Solis et al. 2017[107]^*^ | 1 | 1 | | | 1 | | 1 | | 1 | | 1 | | 1 | | 1 | | 1 | | 1 | | 1 | | 1 | | 12 | | |
| Bai et al. 2016[108] | 1 | 1 | | | 1 | | 1 | | 1 | | 1 | | 1 | | 1 | | 1 | | 1 | | 1 | | 1 | | 12 | | |
| Cui et al. 2016[109]^*^ | 1 | 1 | | | 1 | | 1 | | 1 | | 1 | | 1 | | 1 | | 1 | | 1 | | 0.5 | | 0.5 | | 11 | | |
| Fryer et al. 2016[110] | 1 | 1 | | | 1 | | 1 | | 1 | | 1 | | 1 | | 1 | | 1 | | 1 | | 0.5 | | 1 | | 11.5 | | |
| Gao et al. 2015[111] | 1 | 1 | | | 1 | | 1 | | 1 | | 1 | | 1 | | 1 | | 1 | | 1 | | 1 | | 1 | | 12 | | |
| Gao et al. 2018[112]^*^ | 1 | 1 | | | 1 | | 1 | | 1 | | 1 | | 1 | | 1 | | 1 | | 1 | | 1 | | 1 | | 12 | | |
| Gao et al. 2020[113] | 1 | 1 | | | 1 | | 1 | | 1 | | 1 | | 1 | | 1 | | 1 | | 1 | | 1 | | 1 | | 12 | | |
| Gou et al. 2018[114] | 1 | 1 | | | 1 | | 0.5 | | 1 | | 1 | | 1 | | 1 | | 1 | | 1 | | 1 | | 1 | | 11.5 | | |
| He et al. 2013[91] | 1 | 1 | | | 1 | | 1 | | 1 | | 1 | | 1 | | 1 | | 1 | | 1 | | 1 | | 1 | | 12 | | |
| Hoptman et al. 2010[54] | 1 | 1 | | | 1 | | 1 | | 1 | | 1 | | 1 | | 1 | | 1 | | 1 | | 1 | | 1 | | 12 | | |
| Hu et al. 2016[86] | 1 | 1 | | | 1 | | 1 | | 1 | | 1 | | 1 | | 1 | | 1 | | 1 | | 1 | | 1 | | 12 | | |
| Huang et al. 2010[92] | 1 | 1 | | | 1 | | 1 | | 1 | | 1 | | 1 | | 1 | | 1 | | 1 | | 1 | | 1 | | 12 | | |
| Jin et al. 2021[115] | 1 | 1 | | | 1 | | 1 | | 1 | | 1 | | 1 | | 1 | | 1 | | 1 | | 1 | | 1 | | 12 | | |
| Lei et al. 2015[116] | 1 | 1 | | | 1 | | 1 | | 1 | | 1 | | 1 | | 1 | | 1 | | 1 | | 1 | | 1 | | 12 | | |
| Li et al. 2016[117] | 1 | 1 | | | 1 | | 1 | | 1 | | 1 | | 1 | | 1 | | 1 | | 1 | | 1 | | 1 | | 12 | | |
| Li et al. 2017[118]^*^ | 1 | 1 | | | 1 | | 1 | | 1 | | 1 | | 1 | | 1 | | 1 | | 1 | | 1 | | 1 | | 12 | | |
| Lian et al. 2018[119] | 1 | 1 | | | 1 | | 1 | | 1 | | 1 | | 1 | | 1 | | 1 | | 1 | | 1 | | 1 | | 12 | | |
| Liu et al. 2016[120] | 1 | 1 | | | 1 | | 1 | | 1 | | 1 | | 1 | | 1 | | 1 | | 1 | | 1 | | 1 | | 12 | | |
| Liu et al. 2006[121] | 1 | 1 | | | 1 | | 1 | | 1 | | 1 | | 1 | | 1 | | 1 | | 1 | | 1 | | 0.5 | | 11.5 | | |
| Liu et al. 2018[93]^*^ | 1 | 1 | | | 0.5 | | 1 | | 1 | | 1 | | 1 | | 1 | | 1 | | 1 | | 0.5 | | 1 | | 11 | | |
| Liu et al. 2018[122] | 1 | 1 | | | 1 | | 1 | | 1 | | 1 | | 1 | | 1 | | 1 | | 1 | | 0.5 | | 1 | | 11.5 | | |
| Lui et al. 2010[85] | 1 | 1 | | | 1 | | 1 | | 1 | | 1 | | 1 | | 1 | | 1 | | 1 | | 1 | | 1 | | 12 | | |
| Lui et al. 2015[34] | 1 | 1 | | | 1 | | 1 | | 1 | | 1 | | 1 | | 1 | | 1 | | 1 | | 1 | | 1 | | 12 | | |
| Ren et al. 2013[35] | 1 | 1 | | | 1 | | 1 | | 1 | | 1 | | 1 | | 1 | | 1 | | 1 | | 1 | | 1 | | 12 | | |
| Salvador et al. 2017[123] | 1 | 1 | | | 1 | | 1 | | 1 | | 1 | | 1 | | 1 | | 1 | | 1 | | 1 | | 1 | | 12 | | |
| Shan et al. 2021[124]^*^ | 1 | 1 | | | 1 | | 0.5 | | 1 | | 1 | | 1 | | 1 | | 1 | | 1 | | 1 | | 1 | | 11.5 | | |
| Tang et al. 2019[125] | 1 | 1 | | | 1 | | 1 | | 1 | | 1 | | 1 | | 1 | | 1 | | 1 | | 0.5 | | 1 | | 11.5 | | |
| Turner et al. 2013[57] | 1 | 1 | | | 0.5 | | 1 | | 1 | | 1 | | 1 | | 1 | | 1 | | 1 | | 1 | | 0.5 | | 11 | | |
| Wang et al. 2019[126] | 1 | 1 | | | 0.5 | | 1 | | 1 | | 1 | | 1 | | 1 | | 1 | | 1 | | 1 | | 1 | | 11.5 | | |
| Wu et al. 2019[127] | 1 | 1 | | | 1 | | 1 | | 1 | | 1 | | 1 | | 1 | | 1 | | 1 | | 1 | | 1 | | 12 | | |
| Xie et al. 2021[128] | 1 | 1 | | | 0.5 | | 1 | | 1 | | 1 | | 1 | | 1 | | 1 | | 1 | | 1 | | 1 | | 11.5 | | |
| Yan et al. 2020[50] | 1 | 1 | | | 1 | | 1 | | 1 | | 1 | | 1 | | 1 | | 1 | | 1 | | 1 | | 1 | | 12 | | |
| Yang et al. 2021[129] | 1 | 1 | | | 1 | | 1 | | 1 | | 1 | | 1 | | 1 | | 1 | | 1 | | 1 | | 1 | | 12 | | |
| Yang et al. 2019[130] | 1 | 1 | | | 1 | | 1 | | 1 | | 1 | | 1 | | 1 | | 1 | | 1 | | 0.5 | | 1 | | 11.5 | | |
| Yu et al. 2014,2013[55, 56] | 1 | 1 | | | 1 | | 1 | | 1 | | 1 | | 1 | | 1 | | 1 | | 1 | | 1 | | 1 | | 12 | | |
| Zhao et al. 2019[131] | 1 | 1 | | | 1 | | 1 | | 1 | | 1 | | 1 | | 1 | | 1 | | 1 | | 1 | | 1 | | 12 | | |
| Zheng et al. 2016[132] | 1 | 1 | | | 1 | | 1 | | 1 | | 1 | | 1 | | 1 | | 1 | | 1 | | 1 | | 1 | | 12 | | |
| Zhou et al. 2019[133]^*^ | 1 | 1 | | | 0.5 | | 1 | | 1 | | 1 | | 1 | | 1 | | 1 | | 1 | | 1 | | 1 | | 12 | | |

Items 1 to 12 refers to:

1. Participants were evaluated prospectively, specific diagnostic criteria were applied, and demographic data was reported.

2. Healthy comparison subjects were evaluated prospectively, psychiatric and medical illnesses were excluded, and demographic data was reported.

3. Important variables (e.g. clinical scales, illness history, number of episodes; drug status, non-drug therapy status) were checked either by stratification or statistically.

4. Sample size per group > 10, and no significant difference in age and sex existed.

5. Magnet strength at least 1.5T.

6. Whole brain analysis was automated with no a-priori regional selection.

7. Coordinates reported in a standard space (in condition of no significant differences between groups, this item would also be regarded met since no coordinates need to be reported).

8. The imaging technique used was clearly described so as it could be reproduced.

9. Measurements were clearly described so that they could be reproduced.

10. Results have been corrected for multiple comparison

11. Statistical parameters for significant and important non-significant differences were provided.

12. Conclusions were consistent with the results obtained and the limitations were discussed.

**Table S2. First-level uncorrected results: Insomnia>HC (p<.005; k≥20 mm^3^)**

| Volume(mm3) | BA | Hemisphere | Label | MNI coordinate | | |
| --- | --- | --- | --- | --- | --- | --- |
|  |  |  |  | x | y | z |
| 1176 | 20 | L | Fusiform Gyrus | -44 | -34 | -18 |
|  | 36 | L | Parahippocampal Gyrus | -42 | -38 | -10 |
| 648 | 29 | L | Posterior Cingulate | -6 | -48 | 6 |
| 648 | 6 | L | Precentral Gyrus | -30 | -6 | 54 |
| 560 | 32 | R | Anterior Cingulate | 12 | 42 | 21 |
| 560 | 4 | R | Precentral Gyrus | 32 | -12 | 48 |
| 560 | 6 | R | Precentral Gyrus | 18 | -12 | 62 |
| 480 |  | R | Lentiform Nucleus | 24 | -9 | -6 |
| 456 | 31 | R | Precuneus | 16 | -56 | 40 |
| 448 | 21 | L | Middle Temporal Gyrus | -57 | -3 | -27 |
| 448 |  | L | Claustrum | -36 | 1 | -5 |
| 448 | 29 | R | Posterior Cingulate | 8 | -56 | 14 |
| 448 | 18 | L | Cuneus | -3 | -90 | 21 |
| 448 | 30 | L | Posterior Cingulate | -12 | -62 | 20 |
| 448 | 2 | R | Postcentral Gyrus | 54 | -27 | 39 |
| 392 | 19 | R | Middle Occipital Gyrus | 57 | -63 | -3 |
| 320 | 11 | R | Superior Frontal Gyrus | 18 | 60 | -22 |
| 224 | 25 | L | Medial Frontal Gyrus | -9 | 13 | -23 |
| 200 | 19 | L | Superior Occipital Gyrus | -32.7 | -86 | 32.7 |
| 136 | 18 | L | Middle Occipital Gyrus | -30 | -96 | 10 |
| 48 | 19 | R | Superior Occipital Gyrus | 36 | -86 | 28 |

BA=Brodmann Area; MNI=Montreal Neurological Institute; HC=healthy control; R=right; L=left.

**Table S3. First-level uncorrected results: SCZ>HC (p<.005; k≥20 mm^3^)**

| Volume(mm3) | BA | Hemisphere | Label | MNI coordinate | | |
| --- | --- | --- | --- | --- | --- | --- |
|  |  |  |  | x | y | z |
| 2840 |  | R | Caudate | 16 | 2 | 12 |
|  |  | R | Caudate | 12 | 12 | 8 |
|  |  | R | Lentiform Nucleus | 26 | -4 | 10 |
|  |  | R | Lentiform Nucleus | 24 | 12 | 8 |
| 2216 |  | L | Lentiform Nucleus | -16 | 12 | -2 |
|  |  | L | Lentiform Nucleus | -18 | 8 | -12 |
|  |  | L | Caudate | -4 | 14 | 6 |
| 1768 | 13 | R | Insula | 44 | 20 | 10 |
|  | 13 | R | Insula | 40 | 28 | 12 |
|  | 45 | R | Inferior Frontal Gyrus | 50 | 26 | 6 |
| 1528 | 13 | L | Insula | -42 | 4 | 8 |
|  | 13 | L | Insula | -42 | 20 | 12 |
| 1240 |  | R | Lentiform Nucleus | 22 | 14 | -6 |
|  |  | R | Lentiform Nucleus | 30 | 10 | -12 |
| 1240 |  | L | Lentiform Nucleus | -24 | -2 | 12 |
|  |  | L | Lentiform Nucleus | -26 | -12 | 12 |
|  |  | L | Caudate | -16 | 2 | 18 |
|  |  | L | Caudate | -16 | -6 | 20 |
| 848 | 11 | R | Middle Frontal Gyrus | 32 | 42 | -14 |
|  | 11 | R | Middle Frontal Gyrus | 24 | 44 | -20 |
| 808 | 8 | L | Superior Frontal Gyrus | -4 | 36 | 48 |
| 728 | 9 | R | Medial Frontal Gyrus | 4 | 42 | 28 |
| 616 | 46 | L | Inferior Frontal Gyrus | -40 | 42 | -6 |
|  | 47 | L | Inferior Frontal Gyrus | -42 | 38 | -4 |
| 248 | 39 | R | Angular Gyrus | 48 | -70 | 36 |
| 224 | 20 | R | Inferior Temporal Gyrus | 68 | -40 | -20 |
|  | 21 | R | Middle Temporal Gyrus | 68 | -34 | -16 |
| 208 |  | L | Parahippocampal Gyrus | -30 | -22 | -24 |
| 160 | 8 | R | Superior Frontal Gyrus | 28 | 44 | 42 |
| 128 |  | R | Claustrum | 34 | 2 | 8 |
| 112 | 13 | R | Insula | 44 | 2 | 12 |
| 104 | 44 | R | Precentral Gyrus | 48 | 8 | 6 |
| 96 |  | L | Parahippocampal Gyrus | -30 | -10 | -16 |
| 96 | 47 | R | Inferior Frontal Gyrus | 44 | 36 | -8 |
| 96 | 13 | L | Inferior Frontal Gyrus | -44 | 30 | 6 |
| 88 | 20 | L | Inferior Temporal Gyrus | -52 | -6 | -44 |
| 88 | 40 | L | Inferior Parietal Lobule | -48 | -50 | 42 |
| 80 | 20 | L | Inferior Temporal Gyrus | -54 | -28 | -18 |
| 80 |  | L | Hypothalamus | -4 | -6 | -12 |
| 80 | 17 | R | Lingual Gyrus | 18 | -93 | 0 |
| 80 |  | L | Lentiform Nucleus | -32 | -2 | 2 |
| 80 | 46 | R | Inferior Frontal Gyrus | 48 | 42 | 2 |
| 80 |  | L | Thalamus | -6 | -14 | 12 |
| 80 | 9 | R | Medial Frontal Gyrus | 14 | 48 | 12 |
| 80 | 32 | L | Anterior Cingulate | -8 | 36 | 18 |
| 64 |  | R | Cerebellum | 32 | -45 | -28 |
| 64 | 13 | L | Insula | -40 | 10 | -8 |
| 64 | 13 | R | Insula | 48 | 12 | 0 |
| 64 | 19 | R | Middle Occipital Gyrus | 40 | -80 | 10 |
| 64 | 9 | R | Medial Frontal Gyrus | 8 | 56 | 10 |
| 64 | 10 | L | Superior Frontal Gyrus | -24 | 54 | 18 |
| 56 |  | L | Cerebellum | -24 | -42 | -42 |
| 56 | 21 | L | Middle Temporal Gyrus | -56 | 4 | -36 |
| 56 | 36 | L | Parahippocampal Gyrus | -42 | -38 | -14 |
| 56 | 35 | R | Parahippocampal Gyrus | 24 | -18 | -12 |
| 56 | 18 | L | Lingual Gyrus | -36 | -72 | -6 |
| 56 | 10 | R | Supramarginal Gyrus | 26 | 68 | -4 |
| 56 | 21 | L | Middle Frontal Gyrus | -60 | -30 | 0 |
| 56 | 19 | L | Middle Temporal Gyrus | -30 | -66 | 6 |
| 56 | 40 | L | Lingual Gyrus | -60 | -48 | 30 |
| 56 | 9 | R | Supramarginal Gyrus | 12 | 54 | 30 |
| 48 |  | L | Insula | -48 | 12 | -4 |
| 48 | 10 | L | Superior Frontal Gyrus | -18 | 72 | 10 |
| 40 | 34 | R | Uncus | 18 | -8 | -28 |
| 32 | 20 | L | Inferior Temporal Gyrus | -52 | -24 | -32 |
| 32 | 10 | R | Medial Frontal Gyrus | 8 | 66 | 12 |
| 32 | 39 | R | Middle Temporal Gyrus | 44 | -78 | 20 |
| 32 | 2 | R | Postcentral Gyrus | 48 | -26 | 33 |
| 32 | 8 | L | Superior Frontal Gyrus | -12 | 50 | 32 |
| 32 | 7 | L | Precuneus | 0 | -58 | 44 |
| 32 | 40 | L | Inferior Parietal Lobule | -42 | -46 | 48 |

BA=Brodmann Area; MNI=Montreal Neurological Institute; SCZ=schizophrenia; HC=healthy control; R=right; L=left.

**Table S4. First-level uncorrected results: HC>Insomnia (p<.005; k≥20 mm^3^)**

| Volume(mm3) | BA | Hemisphere | Label | MNI coordinate | | |
| --- | --- | --- | --- | --- | --- | --- |
|  |  |  |  | x | y | z |
| 640 | 31 | L | Cingulate Gyrus | 2 | -44 | 36 |
|  | 31 | L | Cingulate Gyrus | -4 | -38 | 36 |
| 352 |  | R | Cerebellum | 14 | -60 | -48 |
| 288 |  | L | Cerebellum | -15 | -57 | -48 |
| 288 |  | R | Cerebellum | 6 | -68 | -46 |
| 288 |  | R | Cerebellum | 12 | -81 | -21 |
| 288 | 37 | L | Fusiform Gyrus | -57 | -63 | -12 |
| 288 |  | R | Cerebellum | 3 | -72 | 3 |
| 288 | 9 | R | Superior Frontal Gyrus | 27 | 51 | 24 |
| 288 | 40 | L | Supramarginal Gyrus | -42 | -39 | 33 |
| 288 | 24 | R | Cingulate Gyrus | 9 | -9 | 36 |
| 288 | 6 | R | Middle Frontal Gyrus | 33 | 3 | 48 |
| 288 | 8 | R | Superior Frontal Gyrus | 9 | 33 | 48 |
| 288 | 7 | L | Precuneus | -2 | -50 | 54 |
| 280 | 38 | L | Superior Temporal Gyrus | -52 | 10 | -22 |
| 264 | 11 | L | Medial Frontal Gyrus | 0 | 30 | -24 |
| 264 | 37 | L | Fusiform Gyrus | -36 | -48 | -12 |
| 264 | 13 | R | Insula | 44 | -14 | 6 |
| 256 | 47 | L | Inferior Frontal Gyrus | -20 | 32 | -22 |
| 256 |  | L | Claustrum | -40 | -14 | 4 |
| 256 |  | L | Thalamus | -2 | -24 | 8 |
| 256 | 19 | L | Middle Occipital Gyrus | -26 | -86 | 16 |
| 224 |  | L | Cerebellum | -36 | -75 | -30 |
| 224 |  | R | Cerebellum | 15 | -42 | -30 |
| 224 | 47 | R | Inferior Frontal Gyrus | 50 | 18 | -12 |
| 224 | 10 | L | Middle Frontal Gyrus | -30 | 51 | 6 |
| 224 | 7 | L | Superior Parietal Lobule | -21 | -42 | 66 |
| 216 | 31 | L | Posterior Cingulate | -24 | -66 | 22 |
| 192 | 10 | R | Middle Frontal Gyrus | 34 | 62 | -8 |
| 168 | 25 | R | Medial Frontal Gyrus | 16 | 14 | -26 |
| 160 | 13 | R | Insula | 48 | 6 | -4 |
| 160 | 32 | L | Anterior Cingulate | -3 | 43 | 8 |
| 160 | 29 | L | Posterior Cingulate | -12 | -48 | 18 |
| 160 | 6 | L | Precentral Gyrus | -39 | -9 | 42 |
| 56 |  | L | Cerebellum | -10 | -98 | -20 |

BA=Brodmann Area; MNI=Montreal Neurological Institute; HC=healthy control; R=right; L=left.

**Table S5. First-level uncorrected results: HC>SCZ (p<.005; k≥20 mm^3^)**

| Volume(mm3) | BA | Hemisphere | Label | MNI coordinate | | |
| --- | --- | --- | --- | --- | --- | --- |
|  |  |  |  | x | y | z |
| 2728 | 24 | L | Anterior Cingulate | -8 | 40 | -8 |
|  | 32 | R | Anterior Cingulate | 6 | 42 | -16 |
|  | 11 | L | Medial Frontal Gyrus | -8 | 50 | -26 |
|  | 11 | L | Medial Frontal Gyrus | -4 | 40 | -20 |
|  | 10 | R | Medial Frontal Gyrus | 14 | 46 | -24 |
| 1816 | 7 | R | Precuneus | 6 | -48 | 50 |
|  | 7 | L | Precuneus | -8 | -46 | 48 |
| 1568 | 4 | R | Precuneus | 44 | -12 | 46 |
|  | 3 | R | Postcentral Gyrus | 54 | -14 | 44 |
| 1152 | 5 | R | Paracentral Lobule | 6 | -38 | 64 |
|  | 3 | R | Postcentral Gyrus | 12 | -28 | 72 |
| 1104 |  | R | Thalamus | 14 | -12 | 4 |
| 896 | 6 | L | Sub-Gyral | -24 | 8 | 56 |
| 840 | 3 | L | Postcentral Gyrus | -52 | -14 | 40 |
| 696 | 22 | L | Superior Temporal Gyrus | -66 | -20 | 6 |
|  | 22 | L | Superior Temporal Gyrus | -58 | -18 | 0 |
| 624 | 23 | L | Cuneus | -8 | -76 | 18 |
| 576 |  | L | Cerebellum | -40 | -74 | -18 |
| 560 | 37 | R | Inferior Temporal Gyrus | 50 | -70 | 0 |
| 560 | 37 | L | Middle Occipital Gyrus | -44 | -68 | 8 |
|  | 37 | L | Middle Occipital Gyrus | -44 | -74 | 4 |
| 488 | 40 | R | Inferior Parietal Lobule | 44 | -54 | 48 |
|  | 40 | R | Inferior Parietal Lobule | 48 | -48 | 42 |
| 432 | 18 | R | Cuneus | 20 | -84 | 28 |
|  | 18 | R | Middle Occipital Gyrus | 20 | -90 | 22 |
| 424 | 47 | L | Inferior Frontal Gyrus | -22 | 10 | -20 |
| 416 |  | L | Thalamus | -14 | -18 | 10 |
| 304 | 29 | R | Posterior Cingulate | 4 | -54 | 14 |
| 256 | 10 | L | Medial Frontal Gyrus | -12 | 60 | 8 |
| 248 | 4 | L | Precentral Gyrus | -16 | -28 | 74 |
| 208 |  | L | Cerebellum | -14 | -56 | -40 |
| 192 | 19 | R | Fusiform Gyrus | 34 | -82 | -10 |
| 192 | 24 | L | Cingulate Gyrus | -4 | 2 | 42 |
| 192 | 7 | R | Precuneus | 22 | -56 | 62 |
| 176 |  | L | Thalamus | -8 | -14 | -4 |
| 176 | 9 | R | Inferior Frontal Gyrus | 48 | 12 | 22 |
|  | 9 | R | Middle Frontal Gyrus | 54 | 18 | 22 |
| 144 | 17 | R | Cuneus | 6 | -82 | 14 |
|  | 18 | R | Lingual Gyrus | 12 | -80 | 10 |
| 144 | 46 | L | Middle Frontal Gyrus | -42 | 34 | 12 |
| 112 | 9 | L | Superior Frontal Gyrus | -24 | 48 | 24 |
| 96 | 30 | R | Cuneus | 18 | -68 | 12 |
|  | 30 | R | Cuneus | 14 | -70 | 12 |
| 88 | 19 | R | Cuneus | 18 | -86 | 36 |
| 88 | 3 | R | Postcentral Gyrus | 50 | -18 | 54 |
| 64 | 13 | L | Insula | -38 | -18 | 12 |
| 64 | 39 | L | Superior Temporal Gyrus | -51 | -54 | 30 |
| 64 | 9 | L | Middle Frontal Gyrus | -45 | 21 | 39 |
| 64 | 2 | R | Postcentral Gyrus | 46 | -24 | 44 |
| 56 |  | L | Cerebellum | -14 | -70 | -22 |
| 56 |  | L | Cerebellum | -30 | -78 | -18 |
| 56 | 18 | L | Inferior Occipital Gyrus | -36 | -84 | 0 |
| 56 | 7 | R | Precuneus | 10 | -76 | 56 |
| 48 |  | L | Cerebellum | -10 | -50 | -50 |
| 48 | 10 | L | Medial Frontal Gyrus | 0 | 62 | -18 |
| 48 | 37 | R | Fusiform Gyrus | 52 | -68 | -12 |
| 48 | 7 | R | Precuneus | 20 | -70 | 50 |
| 40 | 9 | L | Medial Frontal Gyrus | -4 | 58 | 18 |
| 40 | 19 | R | Cuneus | 10 | -84 | 38 |
| 32 | 10 | L | Medial Frontal Gyrus | -18 | 62 | -16 |
| 32 | 19 | R | Fusiform Gyrus | 28 | -70 | -6 |
| 32 | 41 | R | Superior Temporal Gyrus | 62 | -15 | 3 |
| 32 | 41 | R | Superior Temporal Gyrus | 56 | -20 | 6 |
| 32 | 13 | R | Insula | 42 | -8 | 16 |
| 32 | 39 | R | Middle Temporal Gyrus | 46 | -64 | 24 |
| 32 | 18 | R | Cuneus | 26 | -84 | 32 |
| 32 | 19 | L | Precuneus | -36 | -74 | 42 |
| 32 | 40 | L | Inferior Parietal Lobule | -42 | -50 | 52 |
| 32 | 7 | L | Precuneus | -30 | -44 | 58 |
| 32 | 3 | R | Postcentral Gyrus | 24 | -28 | 58 |
| 32 | 7 | L | Precuneus | 0 | -54 | 64 |
| 32 | 5 | L | Postcentral Gyrus | -18 | -38 | 72 |
| 24 | 6 | R | Superior Frontal Gyrus | 24 | 12 | 62 |

BA=Brodmann Area; MNI=Montreal Neurological Institute; SCZ=schizophrenia; HC=healthy control; R=right; L=left.

**Table S6. Subgroup Analysis: significant clusters demonstrating the overlapping and distinct brain activation regions between insomnia and FES or long-term illness SCZ**

| Volume(mm3) | BA | Hemisphere | Label | MNI coordinate | | |
| --- | --- | --- | --- | --- | --- | --- |
|  |  |  |  | x | y | z |
| ***FES*** | | | | | | |
| **A.Significantly greater increased activities in insomnia vs. FES** | | | | | | |
| 24 | 23 | R | Posterior Cingulate | 9.3 | -54.7 | 18 |
|  |  |  |  |  |  |  |
| **B.Significantly greater increased activities in FES vs. insomnia** | | | | | | |
| 1520 |  | L | Putamen, Caudate Body | -22.3 | 3.4 | 12.3 |
|  |  |  |  | -22 | -2 | 14 |
|  |  |  |  | -16 | 0 | 12 |
|  |  |  |  | -28 | -6 | 14 |
|  |  |  |  | -18 | -3 | 15 |
|  |  |  |  | -30 | -11.3 | 12.7 |
|  |  |  |  | -24.9 | -13.1 | 12 |
| 1024 |  | R | Caudate Body, Putamen, Thalamus | 14.9 | 7.8 | 10.1 |
|  |  |  |  | 32 | 6 | 8 |
|  |  |  |  | 24 | 1 | 9 |
|  |  |  |  | 20 | -2 | 12 |
|  |  |  |  | 24 | -2 | 14 |
|  |  |  |  | 10 | 14 | 10 |
|  |  |  |  | 30 | 2 | 6 |
| 672 |  | L | Putamen, Lateral Globus Pallidus, Caudate Body and Caudate Head | -20 | 12 | 2 |
|  |  |  |  | -15 | 10.6 | 0.7 |
| 472 |  | R | Putamen, Caudate Head | 22 | 14.4 | -4 |
|  |  |  |  | 18 | 12 | -4 |
|  |  |  |  | 24 | 14 | -8 |
|  |  |  |  | 14.7 | 12 | -7.3 |
| 264 |  | R | Putamen | 23.6 | 11.7 | 9 |
| 32 | 13 | L | Insula | -40.5 | -14.5 | 16.5 |
|  |  |  |  |  |  |  |
| **C.Overlapping decreased activities (HC > insomnia and HC> FES)** | | | | | | |
| 16 |  | L | Thamlamus, Pulvinar | -4 | -24 | 8 |
| 16 | 31 | L | Cingulate Gyrus | 0 | -44 | 38 |
| 16 | 31 | L | Cingulate Gyrus | 2 | -44 | 40 |
| 8 | 31 | L | Cingulate Gyrus | -2 | -44 | 36 |
| 8 | 31 | L | Cingulate Gyrus | -2 | -42 | 38 |
|  |  |  |  |  |  |  |
| **D.Significantly greater decreased activities in FES vs. insomnia** | | | | | | |
| 216 | 11 | L | Medial Frontal Gyrus | -9 | 53 | -24.9 |
| 40 | 10 | L | Superior Frontal Gyrus | -17.6 | 61.2 | -15.2 |
|  |  |  |  |  |  |  |
| ***Long-term illness SCZ*** | | | | | | |
| **A.Overlapping increased activities (insomnia > HC and long-term illness SCZ > HC)** | | | | | | |
| 184 | 9 | R | Medial Frontal Gyrus | 12 | 42 | 22 |
| 136 | 36,20 | L | Parahippocampal Gyrus | -42 | -38 | -12 |
|  |  |  | Fusiform Gyrus | -44 | -36 | -16 |
|  |  |  |  |  |  |  |
| **B.Significantly greater increased activities in long-term illness SCZ vs. insomnia** | | | | | | |
| 592 | 13 | R | Insula | 47.7 | 17.8 | 13 |
| 288 | 13 | R | Insula | 46 | 10 | 0 |
|  |  |  |  | 47.5 | 7.5 | 8.5 |
| 288 | 13 | R | Insula | 35.6 | 28.8 | 7.2 |
|  |  |  |  | 38.7 | 30.3 | 8.3 |
| 216 |  | R | Putamen | 27.6 | 15.2 | -2.4 |
|  |  |  |  | 23 | 17.3 | -2.7 |
| 56 | 47 | R | Inferior Frontal Gyrus | 44.3 | 35.7 | -7.4 |
|  |  |  |  |  |  |  |
| **C.Significantly greater decreased activities in long-term illness SCZ vs. insomnia** | | | | | | |
| 1632 | 3,4 | R | Postcentral Gyrus, Precentral Gyrus | 42 | -12 | 47.3 |
|  |  |  |  | 51.8 | -14.4 | 46.4 |
|  |  |  |  | 42 | -10 | 40 |
|  |  |  |  | 56.3 | -15.1 | 38 |
| 184 | 5 | R | Paracentral Lobule | 8 | -39 | 62 |
|  |  |  |  | 6 | -38 | 66 |
| 168 | 2,40 | R | Postcentral Gyrus, Inferior Parietal Lobule | 46.6 | -22 | 44 |
|  |  |  |  | 45 | -26 | 45 |
| 88 | 4 | R | Precentral Gyrus | 54 | -4 | 44 |
| 64 | 7 | R | Precuneus | 20 | -54 | 60 |
|  |  |  |  | 20 | -58 | 62 |
| 40 | 19 | L | Inferior Occipital Gyrus | -36 | -84 | -2 |

BA=Brodmann Area; MNI=Montreal Neurological Institute; FES=first episode schizophrenia; SCZ=schizophrenia; HC=healthy control; R=right; L=left.

**Table S7.** **Control Analysis: significant clusters demonstrating the overlapping and distinct brain activation regions between insomnia and SCZ with only adult participants.**

| Volume(mm^3^) | BA | Hemisphere | Label | MNI coordinate | | |
| --- | --- | --- | --- | --- | --- | --- |
|  |  |  |  | x | y | z |
| **A. Overlapping increased activities (insomnia > HC and SCZ > HC)** | | | | | | |
| 96 | 9 | R | Medial Prerontal Gyrus (mPFC) | 12 | 42 | 22 |
| 48 | 36 | L | Parahippocampal Gyrus | -42 | -38 | -12 |
| **B. Significantly greater increased activities in SCZ vs insomnia** | | | | | | |
| 2264 |  | R | Caudate Body, Putmen | 14.5 | 9.5 | 12 |
|  |  |  |  | 18 | 6 | 14 |
|  |  |  |  | 14.9 | 8.9 | 6.2 |
|  |  |  |  | 11 | 5 | 10 |
|  |  |  |  | 20.5 | 4.5 | 8.5 |
|  |  |  |  | 10.5 | 14.3 | 5.8 |
|  |  |  |  | 15.7 | 3.3 | 19.7 |
| 720 |  | L | Putamen, Caudate Body, Lateral Globus Pallidus | -14 | 7.3 | -1.3 |
|  |  |  |  | -18 | 10 | 2 |
|  |  |  |  | -20 | 14 | 0 |
|  |  |  |  | -13.3 | 10 | 3.3 |
|  |  |  |  | -6.7 | 12.7 | 4 |
| 672 |  | R | Putamen | 26 | 14 | 0 |
|  |  |  |  | 22 | 14 | 0 |
|  |  |  |  | 21 | 12 | -4 |
| 488 | 13,45 | R | Insula, Inferior Frontal Gyrus | 42 | 20 | 6 |
|  |  |  |  | 44.7 | 16 | 9.3 |
|  |  |  |  | 41.3 | 20.4 | 12.5 |
|  |  |  |  | 52 | 18 | 12 |
| 488 |  | L | Putamen | -22 | 2 | 11 |
|  |  |  |  | -24 | -2 | 14 |
| 96 |  | R | Putamen | 25 | 11 | 9 |
| 56 |  | R | Putamen | 32 | 4 | 8 |
| 40 |  | L | Caudate Body | -15.6 | 2.4 | 16 |
| **D. Significantly greater decreased activities in insomnia vs SCZ** | | | | | | |
| 384 | 31  31 | L  L | Cingulate Gyrus | -6.8  0.3 | -38.4  -43.3 | 37.6  36.2 |
| **E. Significantly greater decreased activities in SCZ vs insomnia** | | | | | | |
| 232 | 11 | L | Orbital Frontal Cortex (OFC) | -7.5 | 52.3 | -23.1 |

BA=Brodmann Area; MNI=Montreal Neurological Institute; FES=first episode schizophrenia; SCZ=schizophrenia; HC=healthy control; R=right; L=left.

**Table S8 Control Analysis: significant clusters demonstrating the overlapping and distinct brain activation regions between insomnia and SCZ nvolving participants whose ages are above 30.**

| Volume(mm^3^) | BA | Hemisphere | Label | MNI coordinate | | |
| --- | --- | --- | --- | --- | --- | --- |
|  |  |  |  | x | y | z |
| **A. Overlapping increased activities (insomnia > HC and SCZ > HC)** | | | | | | |
| 112 | 36 | L | Parahippocampal Gyrus | -42 | -38 | -12 |
| **B. Significantly greater increased activities in SCZ vs insomnia** | | | | | | |
| 520 |  | R | Caudate Body, Putmen | 16 | 10 | 14 |
|  |  |  |  | 13 | 4 | 13.5 |
|  |  |  |  | 14.8 | 6 | 8.8 |
|  |  |  |  | 12 | 6 | 18 |
|  |  |  |  | 20 | 5 | 9 |
| 128 | 13 | R | Insula | 48.3 | 17.5 | 11.1 |
| **C. Significantly greater decreased activities in SCZ vs insomnia** | | | | | | |
| 1744 | 3,4 | R | Postcentral and Precentral Gyrus | 44 | -12 | 48 |
|  |  |  |  | 52.5 | -15.7 | 46.1 |
|  |  |  |  | 47.2 | -26 | 56 |
| 192 | 5 | R | Paracentral Lobule | 8 | -40 | 66 |
|  |  |  |  | 4 | -39 | 66 |
|  |  |  |  | 8 | -38 | 62 |
| 168 | 2,40 | R | Postcentral Gyrus and Inferior Parietal Lobule | 47 | -22 | 44 |
|  |  |  |  | 46 | -26 | 44 |
| 128 | 4 | R | Precentral Gyrus. | 54 | -6 | 46 |
|  |  |  |  | 54 | -0.5 | 46.5 |

BA=Brodmann Area; MNI=Montreal Neurological Institute; FES=first episode schizophrenia; SCZ=schizophrenia; HC=healthy control; R=right; L=left.
